# Soft Sensing of Local Skin Reactions: A Computer Vision Approach Using Deep Model Colour Space Analysis for Objective Patch Test Evaluation

**DOI:** 10.3390/s26206599

**Published:** 2026-10-17

**Authors:** Dominyka Stragyte, Vaiva Paskeviciute, Vidas Raudonis, Kestutis Stasaitis, Skaidra Valiukeviciene

**Affiliations:** 1 Department of Skin and Venereal Diseases, Lithuanian University of Health Sciences, 50161 Kaunas, Lithuania; dominyka.stragyte@lsmu.lt (D.S.); skaidra.valiukeviciene@lsmu.lt (S.V.); 2 Automation Department, Electrical and Electronics Engineering Faculty, Kaunas University of Technology, 51367 Kaunas, Lithuania; vaiva.paskeviciute@ktu.edu; 3 SustAInLivWork Center of Excellence, Kaunas University of Technology, 51367 Kaunas, Lithuania; 4 Department of Emergency Medicine, The Hospital of Lithuanian University of Health Sciences Kauno klinikos, 50161 Kaunas, Lithuania

**Keywords:** soft sensor, patch testing, deep learning, CIELAB colour space, allergic contact dermatitis, contact dermatitis, medical image segmentation

## Abstract

**Highlights:**

**What are the main findings?**

- The proposed soft sensing pipeline utilizes a hybrid deep learning ensemble to automatically detect and segment healthy skin and patch test markers, minimizing manual diagnostic subjectivity in contact allergy assessment.
- A novel CIELAB colour space analysis establishes a personalized healthy skin baseline for each patient, effectively addressing inter-patient pigment variability.

**What are the implications of the main findings?**

- By calculating the absolute colour deviation, the system reliably categorizes positive allergic reactions into small, medium, and large intensities for objective clinical grading.
- Achieving a peak Dice score of 89.3% and an Intersection over Union (IoU) of 81.3%, the hybrid segmentation ensemble demonstrates a 90% overall diagnostic agreement with expert dermatological assessments.

**Abstract:**

Allergic contact dermatitis (ACD) is a prevalent condition traditionally diagnosed using standardized patch testing, a method limited by subjective visual skin evaluation and delayed skin reaction manifestations. This study introduces a novel “soft optical sensor” methodology that automates the localization and quantification of local skin reactions. The proposed computer vision framework employs a hybrid deep learning ensemble, integrating DeepLabV3+, UperNet, and SegFormer architectures for the precise segmentation of healthy skin and the detection of patch test markers. To objectively evaluate the intensity of patch-tested reactions, the system transforms extracted regions of interest (ROIs) into the CIELAB colour space. A personalized healthy skin baseline is computed for each patient, allowing the measurement of absolute colour deviation to classify skin reactions into three distinct severity categories (small, medium, and large). Evaluated on a clinical dataset, the hybrid ensemble demonstrated high segmentation performance, achieving a peak Dice score of 89.3% and an intersection over union (IoU) of 81.3%. Furthermore, the colour space analysis showed high congruence with a single expert dermatologist’s assessment (90% overall accuracy), particularly in unambiguous reaction extremes. This automated approach offers a scalable, objective, and standardized solution for clinical dermatological evaluations.

## 1. Introduction

Contact allergy represents a delayed Type IV hypersensitivity immune response triggered by environmental allergens. As industrialization, urbanization, and climate change escalate global exposure to novel chemicals and pollutants, the incidence of contact sensitization has surged dramatically. According to recent Global Burden of Disease estimates, dermatitis ranks as the leading contributor to the global burden of skin conditions, with approximately 253 million new cases of contact dermatitis reported in 2021 alone [1]. This high prevalence imposes a severe physical and psychosocial toll on patients, frequently leading to sleep disturbances, anxiety, and a significantly diminished quality of life.

Beyond the individual impact, the global health costs and economic burden associated with contact dermatitis are profound. The economic consequences extend far beyond direct medical expenses, encompassing substantial indirect costs such as absenteeism, presenteeism, and job loss. Studies evaluating the societal costs of chronic dermatitis indicate that the mean total economic burden per patient can reach upwards of $10,000 annually, translating to billions of dollars in societal costs globally. Severe cases often require expensive pharmacological therapies, specialized outpatient care, and occasionally hospitalization, further straining limited healthcare budgets [2].

The primary diagnostic standard for identifying these contact allergens remains the epicutaneous patch test. However, the traditional patch testing methodology is constrained by subjective interpretation, reliance on physician experience, and the necessity for repeated patient visits (typically at 48 h, 72 h, and 7 days). Depending on the intensity of erythema, infiltration, and vesicle formation, dermatologists manually grade positive patch-test reactions into classes, which can introduce subjectivity into the diagnostic process.

This reliance on time-intensive, manual clinical evaluation is becoming increasingly untenable in the face of a severe global shortage of medical specialists. Forecasts from medical organizations predict a critical shortage of up to 124,000 physicians by 2034, with dermatologists representing a rapidly ageing workforce. Currently, patients in many regions face average wait times exceeding 34 days for a dermatology appointment, with rural and underserved populations experiencing even starker disparities. The ageing global population compounds this clinical workload, forcing healthcare systems to prioritize urgent oncological cases over highly debilitating inflammatory conditions. As wait times lengthen, manageable conditions often deteriorate into complex cases requiring intensive interventions.

In this context, the integration of automation and artificial intelligence is not merely an academic pursuit, but a clinical necessity. While previous automated patch-test evaluation systems have made significant strides using single-stage detection architectures (such as YOLO) or standard binary classification networks, a critical research gap remains. Existing diagnostic tools frequently rely on universal colorimetric thresholds or generalized training data, which fail to account for the vast biological variability in human skin pigmentation and baseline vascularity. Consequently, these conventional methods are susceptible to false-positive diagnoses in patients with naturally darker or highly vascularized skin, and false-negative evaluations in exceptionally pale skin.

The soft sensing pipeline proposed in this study joins subjective visual grading and automated digital diagnostics by introducing a highly personalized evaluation strategy. The genuine novelty of this research lies in moving beyond generalized classification to establish a patient-specific benchmark for localized inflammation. Specifically, the main contributions of this work are threefold, such as the development of a hybrid model, the introduction of a tailored CIELAB analysis, and the automated quantification of allergic inflammation. The development of a novel hybrid computer vision framework that integrates DeepLabV3+, UperNet, and SegFormer architectures entails synthesizing these models through a specialized conflict resolution voting algorithm; the system achieves highly precise macroscopic segmentation of healthy skin and fiducial patch test markers, minimizing the false-positive boundary errors common in standalone architectures. The introduction of a tailored CIELAB colour space transformation mathematically isolates the region of interest and computes a personalized healthy skin baseline (*C_baseline_*) for each individual patient. This completely algorithm-driven step effectively mitigates the effects of inter-patient pigment variability and inconsistent clinical lighting.

## 2. State-of-the-Art

In recent decades, significant technological breakthroughs have enabled the automation of diagnostic processes and enhanced their precision within the medical field [3,4]. Computer vision has emerged as one of the most advanced domains where artificial intelligence (AI) technologies are increasingly applied to accurately analyze medical images and recognize various pathological changes [5,6]. In dermatology, computer vision is particularly important, as the diagnosis of skin diseases relies on visual features that can be automatically detected using deep learning methodologies [7,8]. Convolutional neural networks (CNNs) are highly effective in analyzing dermatological images and identifying conditions such as melanoma, psoriasis, and eczema [3,6,8,9].

While deep neural networks dominate automated skin image analysis, advanced diagnostic systems can also be based on multi-component algorithms and sensors [4,5]. Traditional machine learning techniques, including support vector machines (SVM), random forests, or XGBoost, remain highly relevant, especially when the visual dataset is small or when better interpretability is required [10,11,12]. These models classify pre-extracted image feature vectors, such as colour histograms, texture statistics, or the dimensions of reaction zones without demanding massive computational resources [10,11,12]. Furthermore, diagnostics increasingly experiment with multimodal medical signals that provide supplementary information regarding skin tissue physiology [13,14]. Thermographic sensors can capture localized temperature variations linked to inflammatory processes, while moisture loss sensors and skin electrical conductivity tests supply data on electrophysiological changes correlating with immune reactions [13,15]. These signals can be integrated with computer vision results into a unified classification model [13,14].

The equipment utilized in these diagnostic systems varies significantly, ranging from professional, colour-calibrated digital cameras and specialized medical dermatoscopes to consumer-grade devices like smartphones [8,13,16,17]. While smartphone cameras are widely accessible, their wide-angle lenses and autofocus algorithms can introduce geometric distortions, often requiring hardware or software correction [5,8,16]. The synergy of these multimodal technologies forms the foundation for highly reliable automated diagnostic systems that can seamlessly integrate into clinical environments [3,5].

Deep learning algorithms recognize complex image patterns and make decisions based on massive databases [3,7,16]. A primary advantage of this technology is diagnostic accuracy [4,5]. Studies indicate that deep learning models can perform at or above the accuracy level of experienced dermatologists, achieving up to 91% accuracy in detecting melanoma [7,8,18]. Models trained on large datasets have successfully identified common skin lesions with average accuracies reaching 87–95% [3,5,16]. Despite these achievements, challenges remain, primarily concerning data scarcity, dataset diversity, and ethical or legal liability frameworks regarding patient privacy [3,4,19].

In object detection tasks, deep learning architectures are categorized into two-stage and one-stage models [5,9,16]. Two-stage architectures, such as R-CNN and Faster R-CNN, first generate potential object regions and subsequently classify them [5,9]. While they achieve high accuracy, they are computationally heavy and slower [5,9]. Conversely, one-stage architectures like YOLO (You Only Look Once), SSD, and RetinaNet were developed to minimize computational complexity and maximize speed [5,16,20]. These models perform localization and classification in a single neural network pass [16]. The YOLO architecture treats object detection as a regression problem, predicting bounding box coordinates and classes simultaneously [16,20].

In dermatological image analysis, model speed and computational efficiency are as critical as high accuracy. Real-time analysis in clinical decision-support systems accelerates diagnostic insights and reduces specialist workloads [18]. Consequently, one-stage architectures like YOLO have become an attractive choice for medical image segmentation and pathological area detection [20]. YOLO architectures are especially suited for analyzing skin lesions because they simultaneously detect the lesion’s presence and precisely define its boundaries. Unlike pure classification methods, YOLO-based segmentation models provide comprehensive data on the area, shape, and distribution of pathological regions, which is essential for accurate clinical diagnosis of contact allergy and continuous monitoring of positive reactions [16]. Integrating these computer vision techniques establishes a standardized, objective pathway for diagnosing allergic contact dermatitis and other visually assessed conditions, overcoming the subjectivity of traditional manual evaluations [3,4,5,6,7,8,9,21,22,23,24,25,26,27,28,29,30,31,32].

## 3. Materials and Methods

### 3.1. Patch Testing Methodology and Reaction Evaluation

**Figure 1:**
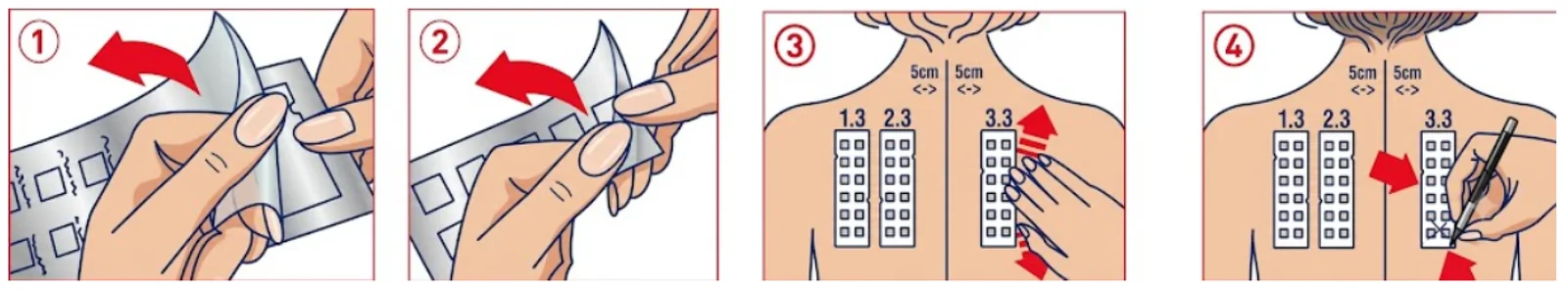
Example of TRUE ^®^ patch-based testing methodology.

The epicutaneous patch test (SmartPractice T.R.U.E. test, Phoenix, AZ, USA) is the primary, standardized diagnostic method used to identify specific allergens and assess a patient’s immune response in cases of allergic contact dermatitis. The testing methodology is based on a controlled contact allergen exposure model. During the procedure, special adhesive patch strips containing various potential environmental and occupational allergens, such as metals (e.g., nickel, cobalt), fragrances, preservatives, and latex, are applied directly to the patient’s skin. These patches are most commonly placed on the patient’s back, an anatomical region chosen to minimize friction and movement-induced artefacts (see Figure 1). To ensure the reliability of the test, the allergens must remain in continuous contact with the skin for an exposure period of 48 to 72 h. Throughout this duration, the patient is required to keep the patches completely intact while strictly avoiding moisture exposure, mechanical irritation, and ultraviolet exposure.

The diagnostic evaluation of patch tests is a multi-step process designed to capture both rapid and delayed immune responses. An initial assessment is typically conducted after the patches are removed to allow physicians to detect early immune reactions. Because allergic contact dermatitis is characterized by a delayed hypersensitivity response, a secondary, final evaluation is important. This follow-up assessment is generally performed 7 days (168 h) post-application to identify delayed immunological reactions that may not have been visible during the initial reading. This prolonged timeline necessitates repeated patient visits, which significantly increases the organizational and time burden on healthcare systems.

**Figure 2:**
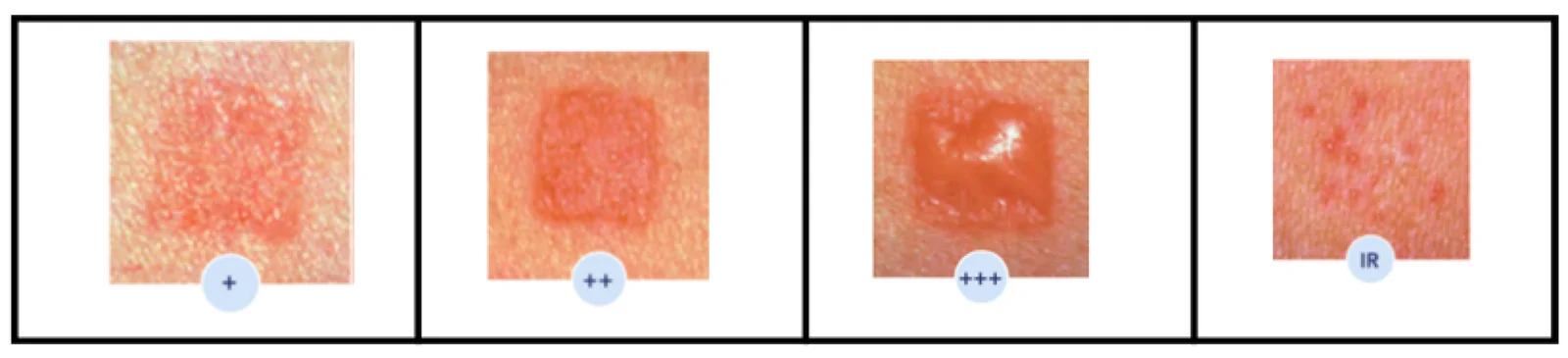
Examples of patch-tested positive skin reactions.

When evaluating the patch testing results, dermatologists analyze specific clinical signs of skin inflammation, primarily focusing on the intensity of erythema (redness), skin infiltration (hardening), and the formation of papules and vesicles. Based on the severity of these inflammatory responses, skin reactions are classified into three main grading categories: A mild allergic response that manifests as erythema, slight skin infiltration (hardening), or the presence of isolated papules (Type 1 reaction (+)). A moderate-intensity allergic response characterized by distinct erythema, noticeable infiltration, papules, and the appearance of isolated vesicles (Type 2 reaction (++)). A severe and highly intense allergic reaction defined by pronounced erythema, deep skin infiltration, and the formation of large, coalescing vesicles (Type 3 reaction (+++)). Irritant reaction: Pustules as well as patchy follicular or homogeneous erythema without infiltrations are usually signs of irritation and do not indicate allergy (IR). Examples of the three main skin-reaction grades are presented in Figure 2.

While patch testing is highly effective and possesses a broad allergen testing spectrum, its traditional manual interpretation presents significant clinical challenges. The evaluation relies entirely on the visual inspection and clinical expertise of the attending physician, introducing a high degree of subjectivity into the diagnostic process. Different specialists may interpret the same skin reactions differently, particularly if the inflammatory response is weak, uneven, or morphologically atypical. Furthermore, non-specific skin irritations, such as general erythema or swelling unrelated to the specific tested allergen, can lead to false-positive or false-negative interpretations. These inherent limitations underscore the critical need for objective, computer vision-based diagnostic tools that are capable of dynamically analyzing subtle colour and textural changes over time to standardize the evaluation of local skin reactions.

### 3.2. Dataset

The experimental dataset for this study consisted of 822 high-resolution digital patch test images collected from 274 unique patients undergoing contact allergy diagnostics at the Hospital of Lithuanian University of Health Sciences Kauno klinikos between 2025 and 2026. Following standardized dermatological protocols, patients were tested with SmartPractice T.R.U.E. Test allergen panels. To accurately track the dynamic kinetics of hypersensitivity responses, exactly three images were captured per patient at specific clinical evaluation intervals: 48 h, 72 h, and 7 days (168 h) post-application. Image acquisition was conducted in natural, uncontrolled clinical environments using a variety of devices, including specialized medical cameras and consumer-grade smartphones (Apple Inc., Cupertino, CA, USA and Samsung Galaxy, Samsung Electronics Co., Ltd., Suwon, South Republic of Korea). This resulted in significant natural variations in lighting, shadowing, contrast, viewpoint, and patient skin tones, which accurately reflect real-world clinical telemedicine conditions but pose a severe challenge for standard computer vision models.

The images were annotated to classify pixels into three distinct, non-overlapping categories, such as (1) the target regions (comprising the black orientation markers), (2) unblemished healthy skin, and (3) the background. The class distribution was highly imbalanced, accurately reflecting the clinical reality. The background class constituted approximately 82% of the total annotated pixels, healthy skin comprised roughly 17.5%, and the critical target markers accounted for only 0.5%.

To facilitate evaluation and minimize data leakage, the dataset was strictly partitioned at the patient level before any patch extraction occurred. All images (from the 48 h, 72 h, and 168 h time points) belonging to a given patient were kept in the exact same subset. The cohort was divided into a training set of 192 patients (576 images), a validation set of 41 patients (123 images), and a completely independent test set of 41 patients (123 images). Due to the high resolution of the clinical photographs, the training and validation images were cropped into smaller 512 × 512-pixel patches prior to model training, maintaining a 50% spatial overlap. After discarding non-informative patches entirely dominated by the background class, this process yielded 23,450 patches for the training set and 5025 patches for the validation set. Because the patch test images from the independent test set were evaluated at their full, uncropped resolution via an automated sliding-window inference protocol, no patches derived from the test set ever appeared in the training or validation sets.

**Figure 3:**
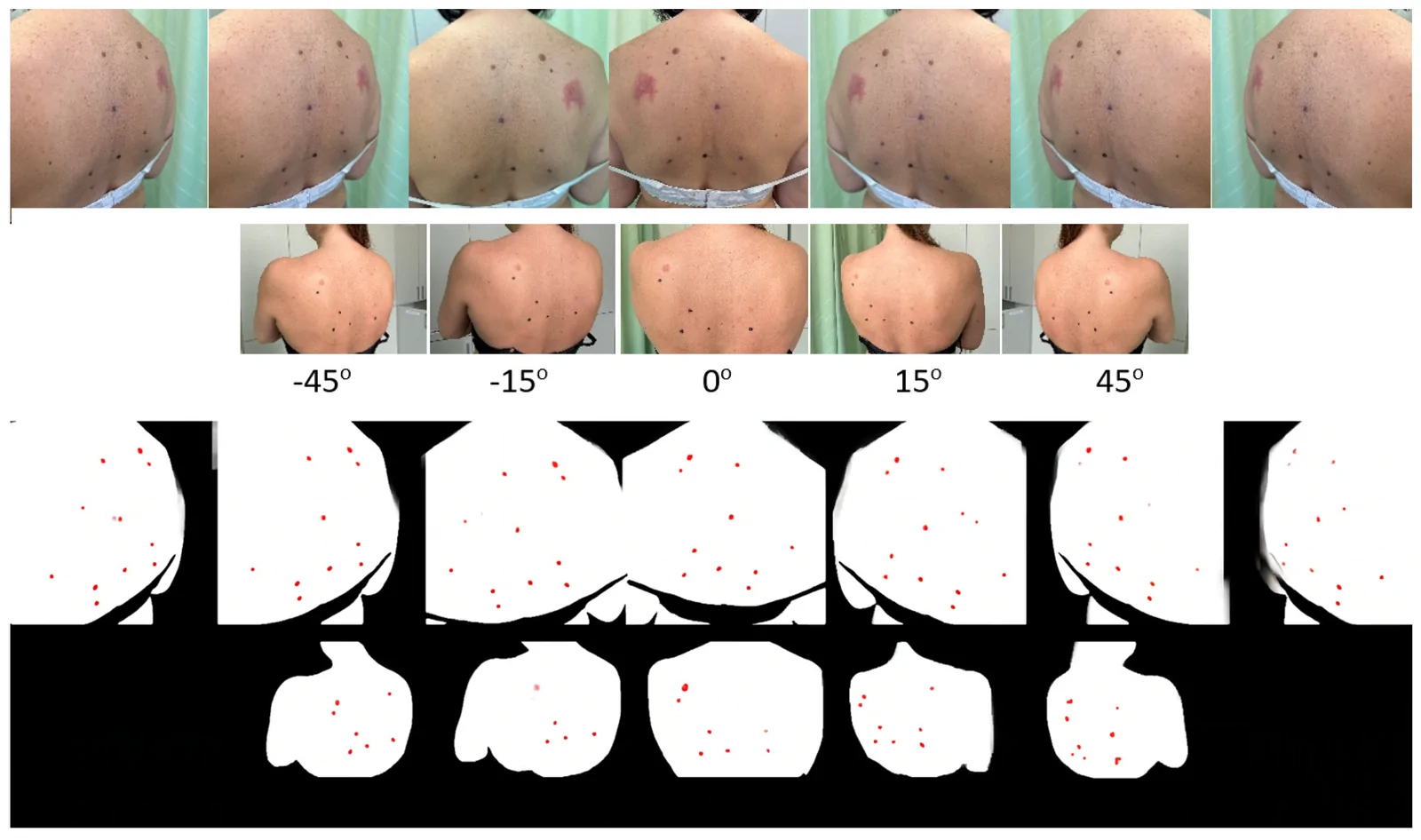
Several examples of images from the raw dataset, where the patient’s back is recorded in different natural camera view angles (ranging from −45° to 45°), lighting conditions, and distances (upper line), alongside the corresponding ground truth segmentation masks of healthy skin and patch markers (lower line). Note that the angles depicted here represent the original photographic viewpoints from the clinic, which are distinct from the −15° to 15° artificial rotations generated later during the data augmentation training phase.

Initial segmentation experiments attempting to use a binary classification approach (isolating only the target areas against a generic background) yielded suboptimal results due to the frequent misclassification of natural skin folds, shadows, and clothing as pathological features. The hierarchical three-class labelling scheme was established to mitigate this limitation and fully leverage the proposed hybrid segmentation architecture. The images were annotated to classify pixels into three distinct, non-overlapping categories, such as (1) the target regions (comprising the black orientation markers), (2) unblemished healthy skin, and (3) the background (see Figure 3). The masks were transformed into three colour images, where target healthy skin was assigned a white colour, dark markers with a red colour, and the background was assigned black (see Figure 3 lower line). Following binarization, contour analysis was performed using an external retrieval mode to identify the primary anatomical region while explicitly eliminating internal noise and small artefacts.

Due to the high resolution of the clinical photographs, feeding entire images directly into the deep learning architectures would exceed standard GPU memory constraints and cause severe contextual downscaling. Therefore, the images were cropped into smaller 512 × 512-pixel patches prior to model training. A 50% spatial overlap was maintained during the patching process to preserve sufficient visual context and prevent boundary information loss. To further alleviate the high class imbalance common in medical imagery, non-informative patches entirely dominated by the background class were discarded.

**Figure 4:**
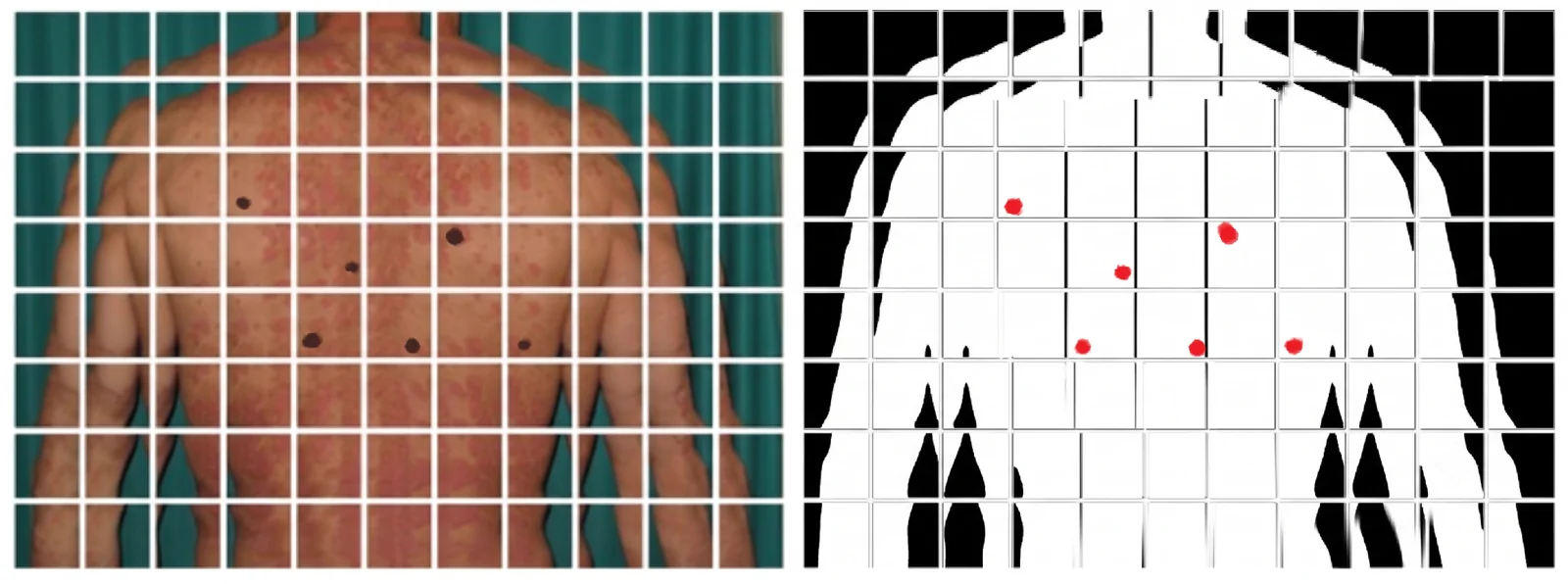
Pre-processed patches before feeding into deep learning models.

To guarantee an unbiased evaluation, the dataset was partitioned at the patient level before any patch extraction occurred. A separate, independent set of full-resolution images was isolated for the testing phase. Because these patch test images were kept completely independent and were evaluated at their full, uncropped resolution via an automated sliding-window inference protocol, no patches derived from the test set ever appeared in the training or validation sets, minimizing the risk of data leakage (see Figure 4).

Finally, to improve the hybrid ensemble model’s generalization capabilities across the diverse clinical imaging conditions, extensive data augmentation was employed. Augmentations were generated on-the-fly during each training iteration and included random horizontal and vertical flipping, random rotations (between −15 and 15 degrees), brightness and contrast adjustments (±0.2 factor), and RGB channel shifts. This dynamic data synthesis effectively expanded the dataset, ensuring the models learned robust feature representations invariant to real-world clinical photography artefacts.

### 3.3. System Architecture and Experimental Setup

**Figure 5:**
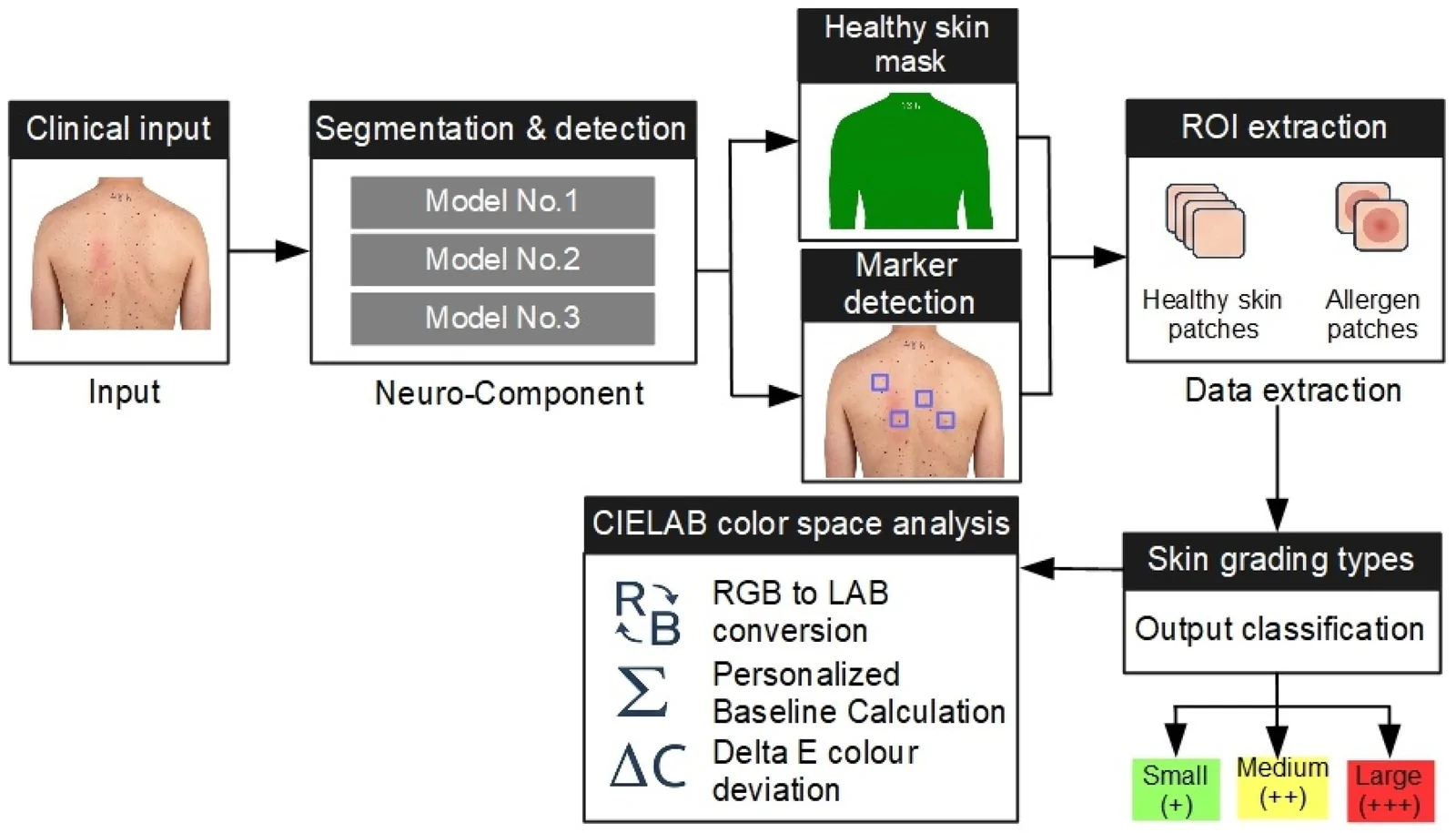
Functional block diagram of proposed skin evaluation system.

We developed a fully automated “soft sensor” architecture to transform this raw optical data into objective diagnostic metrics. The diagnostic pipeline operates in two primary phases, where the AI model identifies medical marker points and segments healthy skin, and the system extracts allergic regions, calculates a personalized baseline, and classifies the intensity of the reaction (see Figure 5).

The first phase relies on a robust hybrid deep learning framework rather than a single standalone algorithm. To accurately navigate the visual complexities of clinical photography, the system integrates three state-of-the-art encoder–decoder architectures (DeepLabV3+, UperNet, and SegFormer). This neural network ensemble initially scans the input image using a hierarchical three-class segmentation scheme to simultaneously classify pixels into patch test markers, unblemished healthy skin, and background elements. A specialized conflict resolution voting algorithm synthesizes the parallel outputs from these three models to generate a highly accurate polygonal mask of the patient’s healthy skin, effectively discarding clothing, skin regions in deep shadow, and background noise.

Following this macroscopic segmentation, the algorithm isolates the black orientation markers (dots) manually drawn on the patient’s skin by medical staff. These fiducial markers are critical; they define the exact spatial coordinates of the allergen patch grid, thereby anchoring the algorithm to the clinically relevant testing zones and drastically reducing the computational search space. Once the anatomical landscape is mapped, the system utilizes the newly generated healthy skin mask to randomly extract 100 unblemished skin fragments per patient, ensuring a statistically representative sample of the individual’s natural skin tone.

The second phase shifts from spatial localization to quantitative evaluation. The system automatically crops the targeted allergen exposure sites bounded by the detected reference markers. Both the healthy skin fragments and the extracted positive reaction regions are then transformed from the standard RGB colour space into the perceptually uniform CIELAB colour space. The system processes the 100 healthy skin samples to compute a personalized, patient-specific statistical baseline *C_baseline_*. This step is important for ensuring diagnostic robustness, as it algorithmically mitigates the effects of varying natural skin pigmentation (melanin concentration) and inconsistent room illumination. Finally, the soft sensor calculates the absolute colour deviation (*D* = |Δ*C*|) for each specific allergen patch against the individualized baseline. By comparing this deviation against mathematically established thresholds, the system classifies the intensity of the allergic reaction into objective categories (types 1, 2 and 3), substantially reducing the subjectivity inherent in manual clinical evaluations.

### 3.4. Selection of the Segmentation Model

The segmentation task in automated patch test evaluation is highly challenging due to the variability of the clinical imaging environment and the extreme class imbalance between the tiny orientation markers, the expansive healthy skin regions, and complex background elements. To achieve accurate segmentation results that can reliably anchor the subsequent CIELAB colorimetric analysis, it is strictly necessary to select the most suitable deep learning models and optimize their parameters. The proposed method utilizes a multi-step selection and refinement process, employing various deep learning architectures and automated hyperparameter optimization via the PyTorch framework and the open-source Optuna library (PyTorch version 2.4.1 was used paired with Python 3.11, CUDA 12.4, and Optuna 4.3.0).

Optuna employs a “define-by-run” programming paradigm, allowing for the dynamic construction of the search space. This is particularly useful for complex experiments involving multiple model types and massive parameter spaces. In this study, the Optuna framework was configured using a tree-structured parzen estimator (TPE) sample and a median pruner. The TPE algorithm effectively leverages previous trial results to focus the hyperparameter search on the most promising areas of the parameter space. The median pruner mechanism was utilized to monitor intermediate results and automatically halt trials if their performance fell below the median of all trials, enabling the efficient exploration of a larger number of parameter combinations within limited computational resources.

The experimental search space was designed to be extensive, evaluating nine diverse segmentation architectures (including U-Net, LinkNet, DeepLabV3+, PSPNet, FPN, PAN, UperNet, and SegFormer) paired with eight different encoder backbones ranging from 8 to 34 million parameters (such as ResNet 34, EfficientNet-b1, Dpn_68, Se_ResNet_50, MobileNet_v2, and Mit_B2). To ensure complete reproducibility, hyperparameter optimization and the training protocol were standardized. The objective function was explicitly defined to maximize the target class Dice score on the validation set. The process involved 405 total trials (exactly 45 trials per architecture). The continuous search space included learning rates (ranging from 1 × 10^−4^ to 1 × 10^−3^), while the discrete search space evaluated batch sizes of 8, 16, and 32. Model training was executed using the AdamW optimizer, minimizing a combined Dice and cross-entropy loss function. The validation protocol utilized the fixed, patient-level validation split. Training was configured for a maximum of 100 epochs, utilizing an early-stopping rule configured to halt training if the validation loss did not improve for 15 consecutive epochs. Finally, a global random seed of 42 was established across all frameworks, and all selected models were initialized using standard pre-trained ImageNet weights to ensure stable and reproducible convergence.

Following this systematic search, three distinct architectures emerged as the top performers and were selected to form the final hybrid ensemble. First, DeepLabV3+, which achieved the highest selection frequency during the trials; second, UperNet, which followed closely as the second most frequently selected architecture; and third, SegFormer, a vision transformer-based architecture that provided a necessary structural contrast to the convolutional models. The exact encoder backbones definitively selected by the Optuna framework were the mix vision transformer (Mit_B2) for DeepLabV3+, the convolutional Dpn_68 for UperNet, and ResNet_34 for SegFormer. To maximize feature extraction capabilities and improve overall model convergence, all final models within the ensemble were initialized using pre-trained ImageNet weights.

### 3.5. Proposed Hybrid Segmentation Model

The literature analysis indicates that complex medical image segmentation tasks frequently benefit from combining multiple distinct architectures into an ensemble system, leveraging the unique strengths of each model to improve overall accuracy and robustness. Given the visual heterogeneity of the human back, varying clinical lighting conditions, and the need to detect tiny reference markers alongside expansive healthy skin, a hybrid segmentation system was constructed for this study.

**Figure 6:**
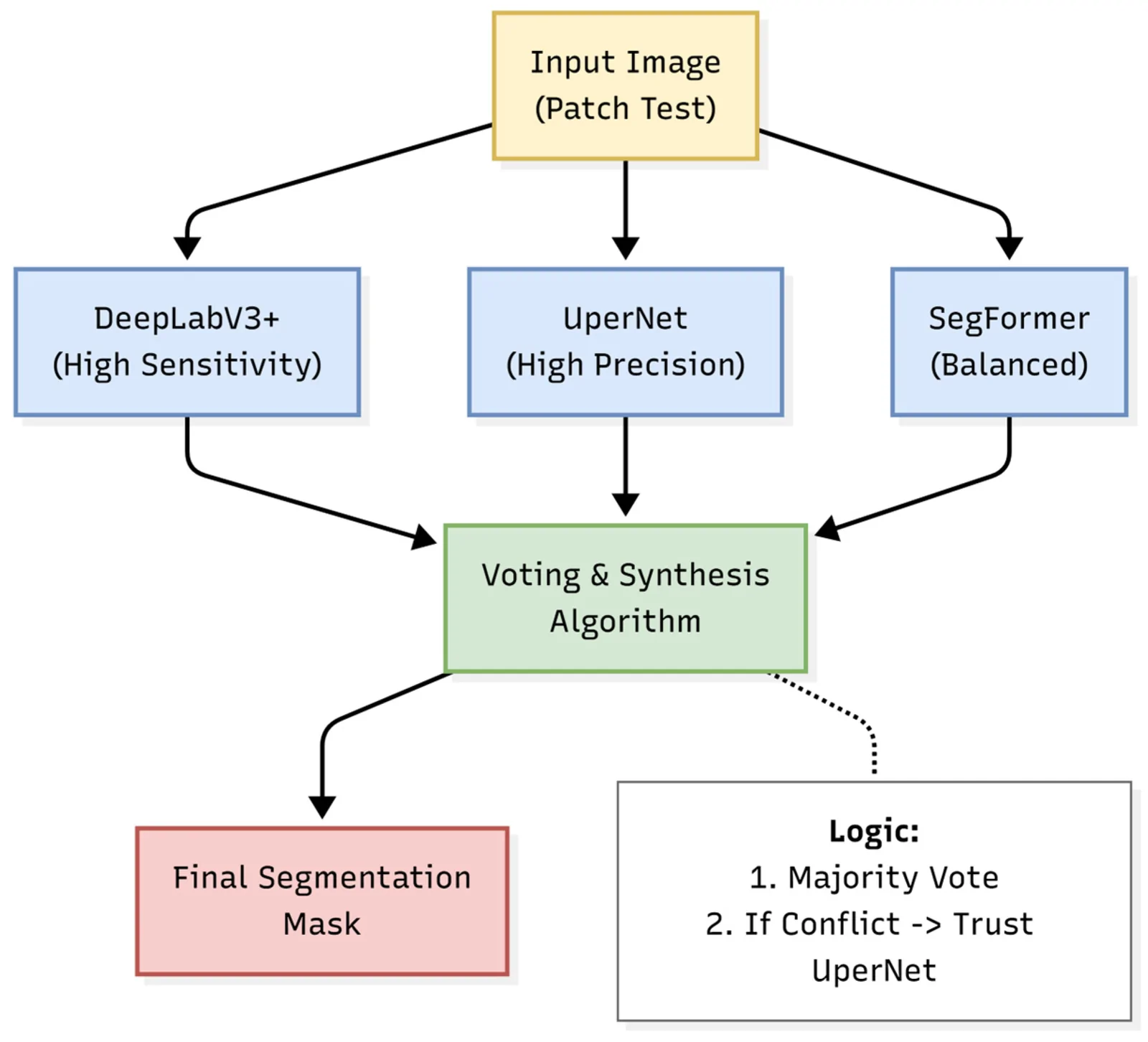
Simplified functional scheme of the proposed hybrid 3-class segmentation model.

The proposed hybrid architecture relies on the parallel execution of the three previously optimized deep learning models: DeepLabV3+, UperNet, and SegFormer (see Figure 6). During the inference phase, each of these models processes the exact same input image and independently generates its own three-class segmentation prediction (patch test markers, healthy skin, and background).

Once the individual predictions are generated, a specialized final mask synthesis algorithm is applied to fuse them into a single, highly accurate segmentation mask. This synthesis relies on a deterministic, pixel-level voting mechanism governed by majority voting and conflict resolution logic. We explicitly note that no individual model weights are applied during this initial voting phase. Each of the three networks contributes an equal, unweighted discrete class prediction. Mathematically, let *C* = {0, 1, 2} represent the set of possible classes (background, healthy skin, patch test markers), and let *p*_1_, *p*_2_, and *p*_3_ ∈ *C* represent the discrete class predictions output by the three models (DeepLabV3+, UperNet, and SegFormer, respectively) for a single pixel. The final assigned class, *P_final_*, is fundamentally determined by the mode function representing the hard vote:

*P_final_* = *mode*(*p*_1_,*p*_2_,*p*_3_) (1)

If at least two out of the three models agree on a classification, that specific class is assigned to the final mask. However, in highly ambiguous cases, such as transition zones or heavy shadows, it is possible for all three models to output a completely different class for the exact same pixel (*p_1_* ≠ *p_2_* ≠ *p_3_*). Under this specific conflict scenario, the algorithm bypasses the standard majority vote and is hardcoded to default to the class predicted by the most reliable individual model. Based on the quantitative evaluation phase, UperNet was designated as the primary reference model for conflict resolution due to its superior baseline precision and low rate of false-positive misclassifications. Therefore, the conflict resolution logic is formulated as:

*P_final_* = *p*_2_ *IF p*_1_ ≠ *p*_2_ ≠ *p*_3_ (2)

**Figure 7:**
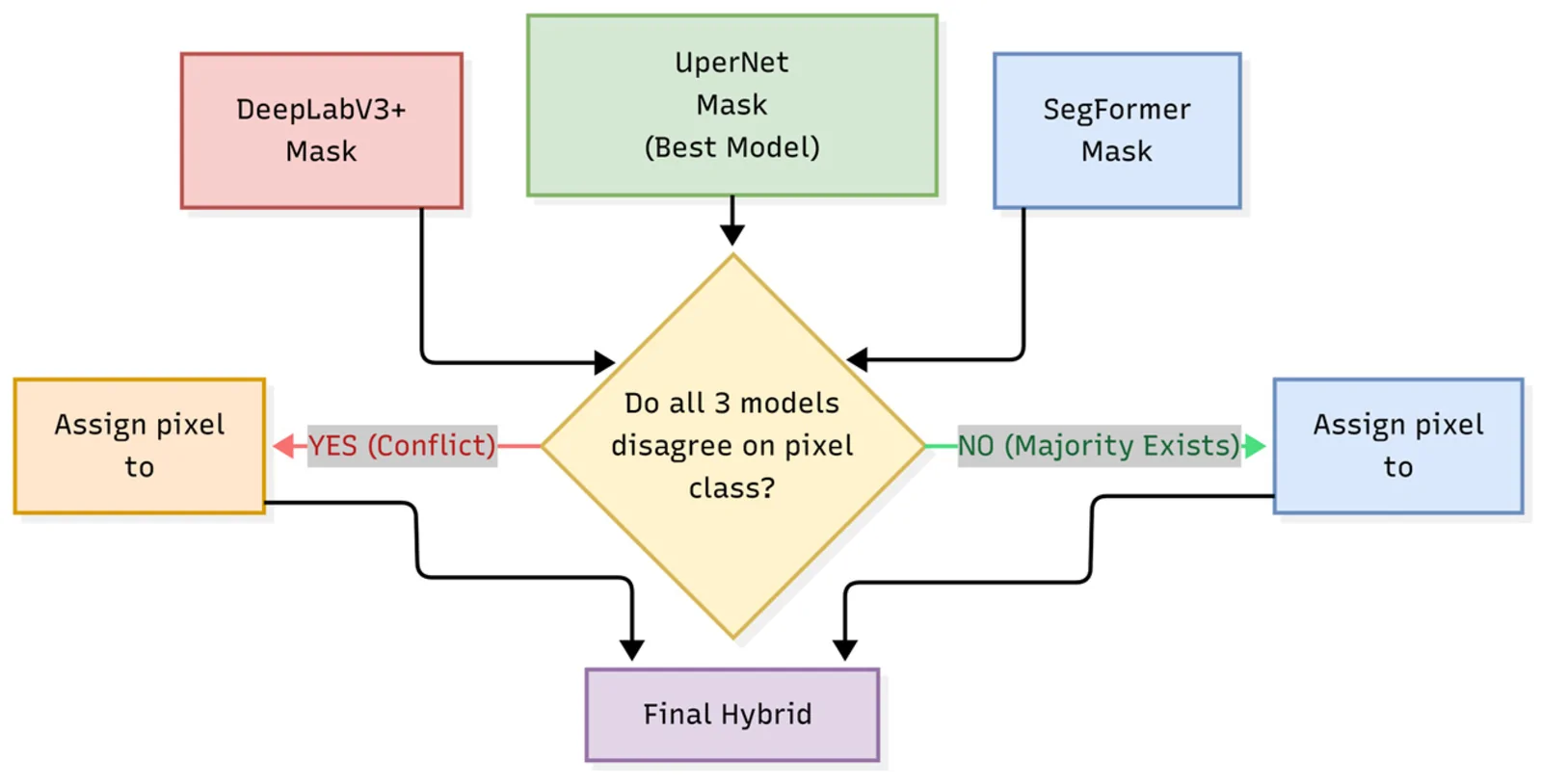
Majority voting and final segmentation map synthesis algorithm.

To empirically justify the selection of unweighted hard voting, an ablation comparison was conducted evaluating this mechanism against soft voting (averaging the continuous softmax probability outputs), weighted soft voting (applying probability weights of 0.4 to UperNet, and 0.3 each to DeepLabV3+ and SegFormer, which were empirically selected based on validation set performance), and the best individual baseline model (UperNet). The proposed hard voting scheme with UperNet conflict resolution achieved the highest target Dice score (89.3%), successfully outperforming unweighted soft voting (88.7%), weighted soft voting (89.1%), and standalone UperNet (88.0%). The discrete hard voting method proved uniquely capable of suppressing ambiguous probability gradients at the complex anatomical boundaries of the human back, which frequently caused over-segmentation when utilizing the continuous soft-voting regimes. Figure 7 represents majority voting scheme.

This hybrid ensemble approach ensures that the diagnostic pipeline uses DeepLabV3+’s exceptional sensitivity to complex target boundaries, SegFormer’s global contextual awareness across varying scales, and UperNet’s precise localization. By mathematically fusing these predictions through the voting algorithm, the final output successfully minimizes pixel classification errors at the ambiguous borders between healthy skin and background elements. The resulting synthesized mask provides a clean and stable spatial map, which acts as the definitive foundation for the subsequent spatial grid mapping, allergen patch cropping, and personalized CIELAB colorimetric analysis.

The performance of the segmentation models was evaluated using standard computer vision metrics adapted for semantic segmentation. In this study, the primary clinical focus is on two distinct foreground classes, such as the manually drawn black orientation markers and the healthy skin. For the purpose of the summarized target performance metrics (Dice and IoU) reported in the results, the values represent the macro-average of these two specific foreground classes, explicitly excluding the background. The primary metric is the class-specific intersection over union (IoU), also known as the Jaccard index, which evaluates the overlap between predicted masks and ground truth masks:

(3) $${\mathrm{IoU}}_{c}=\frac{\left|P_{c}\cap T_{c}\right|}{\left|P_{c}\cup T_{c}\right|}$$

where *P_c_* represents the number of predicted pixels for class *c*, and *T_c_* is the number of pixels in the ground truth for class *c*. Additionally, the system utilizes the Dice coefficient, which emphasizes the overlap between predicted and actual regions and is highly suitable for small or fragmented objects like reference markers:

(4) $${\mathrm{Dice}}_{c}=\frac{2\times \left|P_{c}\cap T_{c}\right|}{\left|P_{c}\right|+\left|T_{c}\right|}$$

Finally, to account for class imbalances between the small markers, the expansive healthy skin, and the background, the class-specific F1-score is calculated as the harmonic mean of precision and recall:

(5) $${F1}_{c}=2\times \frac{{\mathrm{Precision}}_{c}\times {\mathrm{Recall}}_{c}}{{\mathrm{Precision}}_{c}+{\mathrm{Recall}}_{c}}$$

For all reported metrics (IoU, Dice, and F1-score), the values were calculated individually per image for the specific class and subsequently averaged across the entire test dataset. This per-image averaging procedure accurately reflects real-world clinical performance on an image-by-image basis, and accounts for the slight numerical deviations from the strict mathematical relationship between Dice and IoU that occurs when metrics are calculated from globally pooled pixels.

To reflect the specific clinical priorities of the automated pipeline, a custom weighted F1-score was established before any test evaluations were conducted. This custom metric assigns an 80% weight to the target marker class and 20% to the healthy skin class, explicitly excluding the background. The clinical justification for these specific weights is twofold: first, the precise detection of the small target markers is paramount, as they establish the geometric coordinates for the entire spatial mapping grid. The failure here results in total pipeline failure. Second, the background class (e.g., clothing, room fixtures) holds no diagnostic value for the final colorimetric analysis. However, to ensure full statistical transparency and comparability, standard macro, micro, and class-weighted F1-scores were also calculated across all three classes.

### 3.6. Localization of Patch Test Position

**Figure 8:**
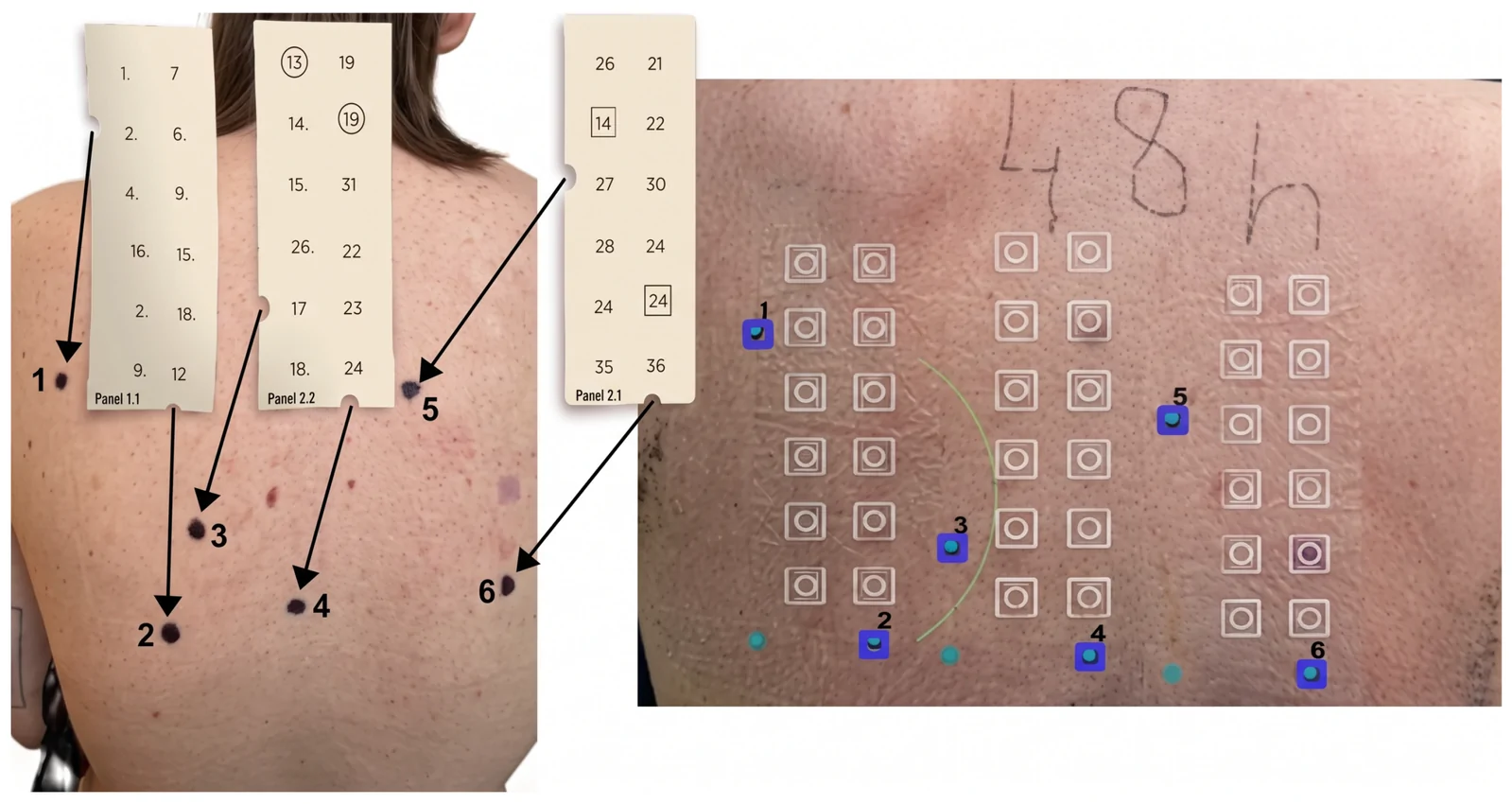
Examples of black markers manually placed by a dermatologist (**left**), and automatically detected real patch positions based on the markers and known geometrical shapes of the patches (**right**).

Once the healthy skin region and the orientation markers are robustly segmented by the hybrid deep learning model, the system proceeds to localize the specific areas where the patch tests were applied (see Figure 8). To achieve this, the algorithm leverages the dedicated marker class from the generated three-class segmentation mask. Because the hybrid model has already isolated these manually drawn dark markers from the background and healthy skin, the system bypasses the need for rudimentary colour mask filters or single-stage regression models.

To refine the spatial data extracted from the segmentation mask, morphological procedures are applied to ensure the structural integrity of the marker contours. Following this, a contour analysis algorithm calculates the geometric parameters of these isolated objects to determine their exact central points. As illustrated in Figure 8 (left), these marker points are used to spatially locate the allergen patches on the skin. However, automated localization is highly challenging because the human back is not a perfectly flat surface. Natural skin folds, anatomical unevenness, and skin elasticity can distort the exact positions and relative distances of these markers, complicating the mapping process. To overcome these biological variables, the algorithm utilizes the known geometric layout of the standardized patch template to calculate the real, hidden patch positions on the skin. As demonstrated in Figure 8 (right), the system relies on the spatial relationship between the centrally detected markers. By computing the precise distance and angle between these specific reference points, the algorithm establishes a stable spatial grid. This geometric calculation maps the exact testing zones (indicated by the white squares), effectively overcoming the challenges of skin elasticity and patient movement. This stable grid perfectly bounds the allergen application sites, allowing the algorithm to automatically and accurately crop the target patches. This process ensures that the region of interest is anatomically relevant and devoid of background noise or clothing, thereby establishing a reliable foundation for the final CIELAB colorimetric evaluation.

**Figure 9:**
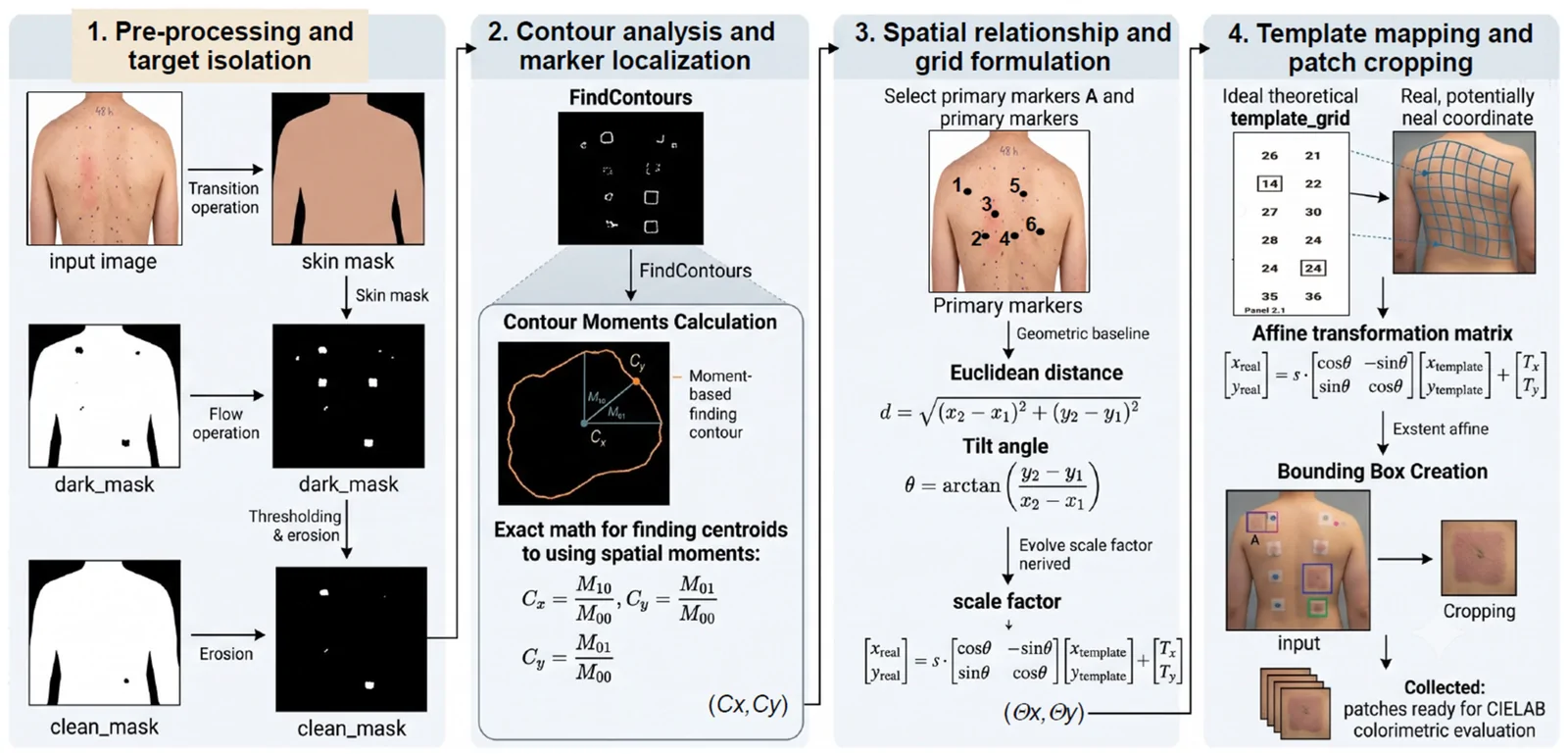
Function diagram of locating the accurate position of the test patch.

The provided Figure 9 illustrates a four-step algorithmic pipeline designed for the automated localization and extraction of allergic reaction patches from a patient’s back. The first phase, “Pre-processing and target isolation”, focuses on separating the relevant regions of interest from the background. The raw input image undergoes targeted operations to generate a segmented skin mask and a preliminary *dark_mask* that captures the manually placed orientation markers. Through precise thresholding and morphological erosion procedures, background noise is effectively eliminated to produce a *clean_mask*, ensuring that only the distinct marker areas remain for processing.

Phase two, “Contour analysis and marker localization”, accurately pinpoints the exact centre of each marker. Using a *FindContours* function, the algorithm isolates the marker boundaries. It then calculates the centroid coordinates (*C_x_*, *C_y_*) by utilizing spatial image moments. The third phase, “Spatial relationship and grid formulation”, establishes a geometric baseline by analyzing the primary detected markers (labelled 1 through 6 on the patient’s skin). The system calculates the Euclidean distance between these primary reference points. This calculation accurately derives the necessary scale factor and rotational data to construct a spatial grid. The final phase, “Template mapping and patch cropping”, aligns an ideal theoretical *template_grid* onto the real, potentially distorted anatomical coordinates. This is achieved using a standard affine transformation. Following this exact spatial transformation, precise bounding boxes are created around the calculated patch locations. The system automatically crops these individual allergen test sites, collecting the isolated patches for the final CIELAB colorimetric evaluation.

### 3.7. Colour Space Analysis

The transition from spatial localization to quantitative diagnostic evaluation requires a robust, mathematically sound representation of optical data. Standard RGB colour spaces are highly susceptible to variations in lighting, and their colour channels are deeply correlated, making them inadequate for precise medical image analysis where subtle pigment shifts dictate clinical decisions. Therefore, the automated soft sensing pipeline transforms the extracted regions of interest (ROIs) from the RGB colour space into the CIELAB colour space. The primary, critical advantage of the CIELAB colour space is its perceptual uniformity. The measured numerical change within the CIELAB coordinate system directly corresponds to a proportionate change in human visual perception. Furthermore, it decouples the luminance from the chromaticity information. This characteristic makes it exceptionally useful for dermatological research, where the severity of a condition is traditionally graded by the human eye based on subtle shifts in skin tone, erythema, and textural swelling. To quantify the allergic reaction, images were transformed from RGB to the perceptually uniform CIELAB colour space. Each pixel *p_i_* is represented as a 3D vector:

*p_i_* = (*L_i_*,*a_i_*,*b_i_*) (6)

where *L_i_* ∈ [0,100] represents the lightness component, *a_i_* denotes the position on the green-to-red colour axis, and *b_i_* denotes the position on the blue-to-yellow colour axis. When analyzing an extracted skin fragment containing *N* pixels, the system aggregates the spatial data by calculating the mean values for each individual channel:

(7) $$\bar{L}=\frac{1}{N}\sum_{i=1}^{N}L_{i},\;\bar{a}=\frac{1}{N}\sum_{i=1}^{N}a_{i},\;\bar{b}=\frac{1}{N}\sum_{i=1}^{N}b_{i}$$

This aggregation effectively smooths the data, mitigating the influence of local image noise, minor textural variations, and small artefacts like body hair or pores, thereby providing a stable mathematical representation of the overall skin state in that specific region. Allergic reactions are primarily characterized by localized inflammation and vasodilation, which visually manifest as a distinct increase in skin redness. Consequently, the diagnostic signal for patch test evaluation is heavily concentrated in the a* (green-red) channel. While traditional colorimetry might employ the standard CIE76 ΔE formula to measure overall colour distances, this research utilizes a task-adapted, modified coefficient specifically optimized to track erythema. Because allergic reactions primarily exhibit an increase in the red channel (a*), a normalized colour coefficient *C* was formulated:

(8) $$C=\frac{\bar{a}}{\bar{L}+\varepsilon }+1$$

In this equation, *a* represents the average redness, $\bar{L}$ represents the average lightness, and ε = 1 serves as a stabilizing constant to prevent potential division by zero or mathematical instability at exceptionally low lightness values. The inclusion of the lightness denominator mathematically normalizes the redness against the overall brightness of the image, thereby compensating for any residual inconsistencies in clinical room illumination or shadowing. For healthy, uninflamed skin, the relationship between the redness and lightness channels typically results in a coefficient value close to 1 (C ≈ 1). In contrast, when the skin is affected by an allergic reaction, the localized erythema drives up the $\bar{a}$ value, resulting in a coefficient greater than 1 (C > 1).

A challenge in automated dermatology is the vast natural diversity of human skin pigmentation. A universal, fixed threshold for “redness” would inevitably fail in diverse clinical populations, triggering false positives in patients with naturally darker or highly vascularized skin, and false negatives in exceptionally pale skin. The proposed soft sensing pipeline overcomes this biological variability through a highly personalized evaluation strategy. Leveraging the previously generated healthy skin mask from the hybrid ensemble model, the algorithm randomly extracts M = 100 fragments of unblemished skin from the patient’s own back.

To mathematically mitigate the spatial correlation inherent to sampling multiple regions from a single continuous image, stringent sampling constraints were applied. Each extracted healthy skin fragment was defined as a 32 × 32-pixel block. The algorithm enforced a strict zero-overlap policy combined with a minimum spatial distance constraint (at least 50 pixels) between the centroids of any two sampled fragments, ensuring a wider, more representative distribution across the anatomical landscape. The system calculates the colour coefficient *C_i_* for each of these patches. To define the personalized statistical baseline *C_baseline_*, the system employs a robust estimator rather than a standard arithmetic mean. By calculating a 20% trimmed mean, the algorithm inherently protects the baseline against localized optical anomalies (for example, a stray freckle, subtle shadowing, or minor skin blemishes) that might have bypassed the masking phase using Formula (9).

(9) $$C_{\mathrm{baseline}}=\frac{1}{M-2k}\sum_{i=k+1}^{M-k}C_{i}$$

In this formulation, *C_i_* represents the sorted colour coefficients in ascending order, and *k* represents the trimming parameter (*k* = 0.2 M). An empirical sensitivity analysis conducted during the validation phase evaluated the stability of this estimator across varying fragment counts (*M* = 25, 50, 75, 100, 150). The analysis demonstrated that the variance of the trimmed mean plateaus consistently beyond 75 fragments; therefore, extracting *M* = 100 fragments safely optimizes the trade-off between computational efficiency and baseline stability. This customized, robust baseline establishes a patient-specific referential benchmark that encodes the individual’s unique melanin concentration and baseline vascularity, substantially reducing inter-patient variability.

This customized baseline establishes a patient-specific referential “ground truth” that inherently encodes the individual’s unique melanin concentration and baseline vascularity. Utilizing a large sample size of 100 patches helps ensure that the baseline is statistically robust against outliers, such as a stray freckle or minor shadow that might have bypassed the masking phase. By establishing this personalized benchmark, the system substantially reduces inter-patient variability, helping to ensure that subsequent measurements evaluate the actual physiological change caused by the allergen rather than the absolute skin colour. Furthermore, it simultaneously accounts for the specific lighting conditions present at the exact moment the photograph was taken, adding a layer of environmental robustness to the analysis. Once the personalized baseline is established, the algorithm processes the cropped ROI for each individual allergen patch. The target patch’s colour coefficient *C*_allergy_ is computed using the same formulation. The final intensity of the allergic reaction is determined by calculating the absolute colour deviation *D*, essentially measuring the magnitude of the shift from the patient’s healthy norm:

*D* = |Δ*C*| = |*C_allergy_* − *C_baseline_*| (10)

This absolute colour deviation *D* serves as a continuous, objective mathematical metric of inflammation. However, to translate this continuous metric into actionable clinical knowledge that aligns with standard dermatological grading systems (such as the +, ++, +++ scale used by physicians), the system categorizes the *D* values using pre-defined threshold limits. These specific values (*T_1_* = 0.05 and *T_2_* = 0.15) were not chosen arbitrarily, nor were they derived from the final test set. To strictly avoid circular validation, these thresholds were statistically established using a one-dimensional k-means clustering procedure applied exclusively to the extracted patches from the training and validation cohorts. By anchoring the cluster centroids to the corresponding expert visual assessments from the training data, the algorithm learned the optimal decision boundaries. The skin reactions are discretely classified into three specific severity grades based on these learned limits:

1. Small reaction: *D* < 0.05 (corresponding to mild erythema and slight infiltration);
2. Medium reaction: 0.05 ≤ *D* < 0.15 (corresponding to distinct erythema and noticeable papules);
3. Large reaction: *D* ≥ 0.15 (corresponding to severe inflammation, deep infiltration, and coalescing vesicles).

Through this structured transformation, the soft sensor pipeline successfully transforms raw pixel data into a standardized medical taxonomy. The subjective, highly variable process of manual visual inspection is augmented by a deterministic, mathematically verifiable algorithm that enhances diagnostic reproducibility across diverse patient demographics and varying clinical environments

## 4. Results

### 4.1. Healthy Skin and Reference Markers Segmentation Performance

To ensure an accurate assessment of the models’ ability to isolate the target regions from healthy skin and background elements, performance was quantified using the Sørensen–Dice coefficient (Dice), the intersection over union (IoU/Jaccard Index), the weighted F1-score, and the Area Under the Curve (AUC). Because the predictions generated by the individual and hybrid models are derived from the exact same paired test images, reporting single performance values is insufficient to establish statistical significance. Therefore, 95% confidence intervals (Cis) were computed for all evaluation metrics utilizing a patient-level bootstrap procedure with 1000 resampling iterations. The comprehensive performance comparison, including these confidence intervals, is detailed in Table 1.

While the custom clinical F1-score successfully highlights the models’ sensitivity to the critical reference markers, standard F1-score variants were also computed across all three classes (target, healthy skin, and background) to provide a complete, unbiased statistical overview. As demonstrated in Table 2, the hybrid ensemble consistently maintains the highest performance across all standard metric variants. Because the background and healthy skin classes dominate the total pixel count of the images, the micro- and class-weighted F1-scores naturally skew very high, further underscoring why the custom 80/20 metric was necessary to accurately evaluate the clinically critical target class.

UperNet architecture demonstrated exceptionally balanced segmentation capabilities, achieving the highest individual Dice score of 88.0% (0.880). Its primary strength lies in its baseline precision and its ability to minimize false-positive misclassifications. The model accurately identified 94.6% of healthy skin pixels and 99.4% of background pixels, with high precision for the markers, meaning only 5.1% of the pixels predicted as the target class were actually healthy skin. In contrast, DeepLabV3+ exhibited the highest sensitivity for target recognition, accurately identifying 92.8% of the target class pixels. However, this sensitivity resulted in a tendency for over-segmentation. The model produced nearly double the false-positive rate of UperNet (9.2%) of its predicted target pixels actually belonged to healthy skin, artificially extending target boundaries into unblemished areas. Consequently, its overall Dice score was the lowest among the three at 83.4% (0.834). The SegFormer architecture occupied an intermediate performance tier, achieving a Dice score of 85.9% (0.859). While it provided a stable global context, it occasionally struggled with boundary precision, incorrectly assigning 8.0% of healthy skin to the target class.

**Figure 10:**
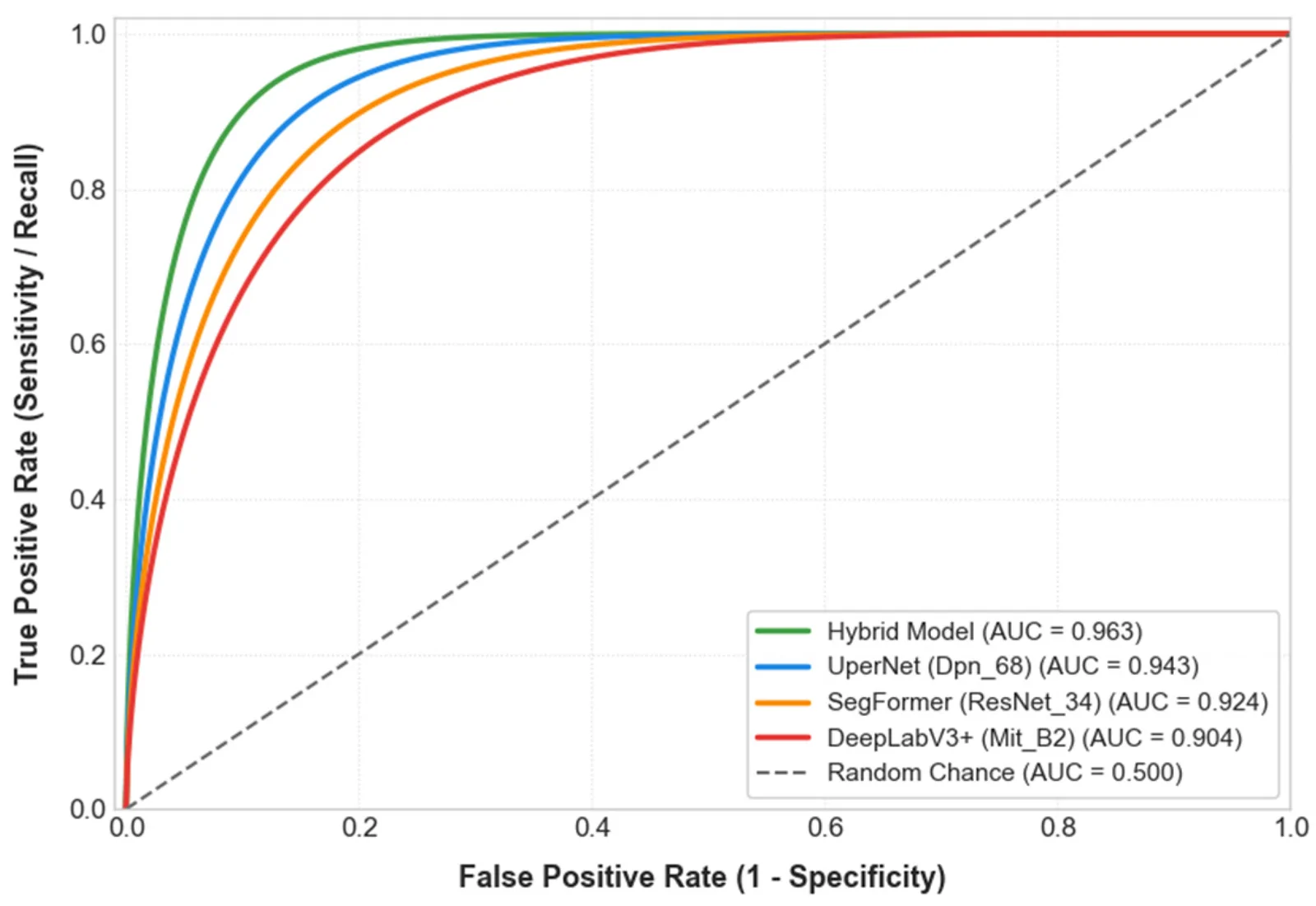
ROC curve of segmentation results.

The receiver operating characteristic (ROC) curve visualizes a model’s segmentation performance by plotting its true positive rate (sensitivity) against its false positive rate (specificity) across various decision thresholds. Because constructing a smooth ROC curve mathematically requires a continuous variable rather than discrete class labels, the curves presented in Figure 10 utilize the raw continuous softmax probability outputs from the individual architectures. For the hybrid model specifically, the continuous score used exclusively for this ROC and AUC analysis was derived by averaging the continuous softmax probability maps of DeepLabV3+, UperNet, and SegFormer, even though the final applied spatial mask relies on the discrete hard voting mechanism previously described. As illustrated in Figure 10, the hybrid model demonstrates the most effective balance of sensitivity and specificity, closest to the top-left corner to achieve the highest overall accuracy and Area Under the Curve (AUC = 0.963).

The proposed hybrid model successfully combined the complementary strengths of these three networks, utilizing DeepLabV3+ for its high sensitivity to small target features, UperNet for its stringent precision and boundary containment, and SegFormer for its balanced contextual awareness. By prioritizing UperNet’s output during conflict resolution voting, the hybrid system actively suppressed false positives while maintaining high detection rates. Quantitative results confirm the superiority of the ensemble approach. The hybrid model outperformed all individual architectures, achieving a peak Dice score of 89.3% (95% CI: 0.882–0.905), marking a 1.3% improvement over the best standalone model. To verify that this performance gain is statistically meaningful rather than a product of random variance, a paired permutation test was applied to the test cohort predictions. The statistical evaluation confirmed a significant difference (*p* < 0.01) between the hybrid ensemble and UperNet, demonstrating that the conflict resolution voting mechanism provides a robust and clinically relevant enhancement in segmentation accuracy. Furthermore, the system achieved an intersection over union (IoU) of 81.3% (0.813) and a weighted F1-score of 90.7% (0.907). The weighted F1-score calculation (assigning an 80% weight to the target class and a 20% weight to healthy skin) proves that the algorithm successfully meets clinical priorities, effectively balancing sensitivity and precision without generating excessive false predictions.

**Figure 11:**
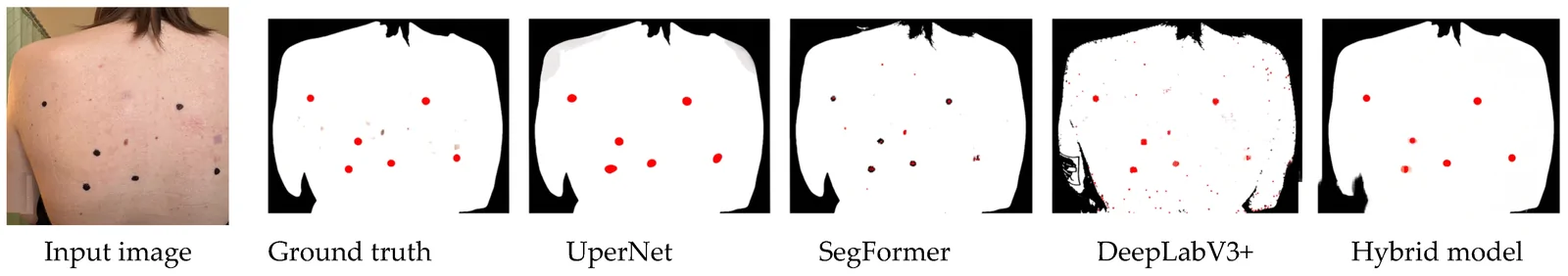
Healthy skin and reference mark segmentation results of all deep models.

**Figure 12:**
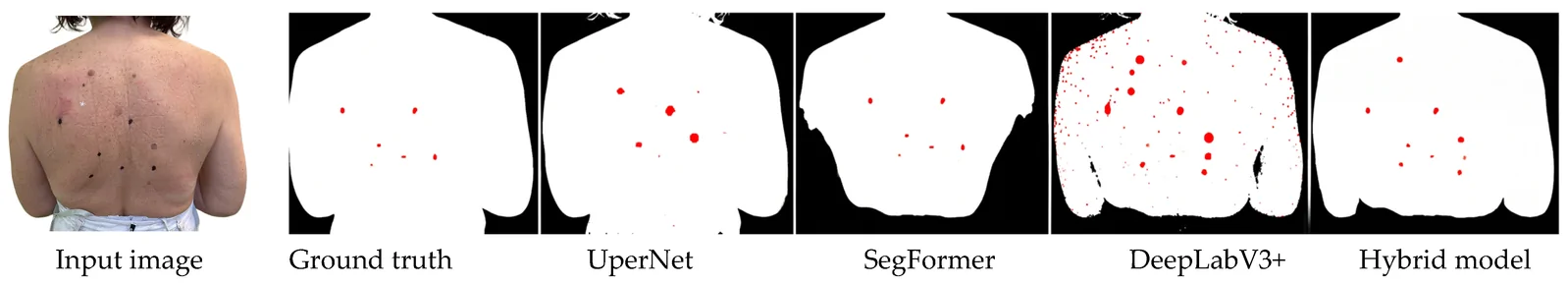
Less successful healthy skin and reference mark segmentation results of all deep models.

Despite the high statistical accuracy, visual analysis of the algorithmic output highlights the remaining challenges inherent to unconstrained dermatological photography (see Figure 11 and Figure 12). A systematic review of the less successful segmentation cases identified four primary sources of error. The models occasionally struggle to define sharp boundaries where the target area gradually fades into healthy skin without a distinct colour or texture gradient. Natural body contours create shadowing that can deceive the algorithms, leading to mask expansion outside the factual boundaries. The presence of body hair, clothing edges, or other external artefacts on the skin surface can introduce localized predictive errors (see Figure 12).

### 4.2. Quantitative Analyses of Allergic Responses and Dermatologist Comparison

#### 4.2.1. Personalized Healthy Skin Baselines and Reaction Variance

**Figure 13:**
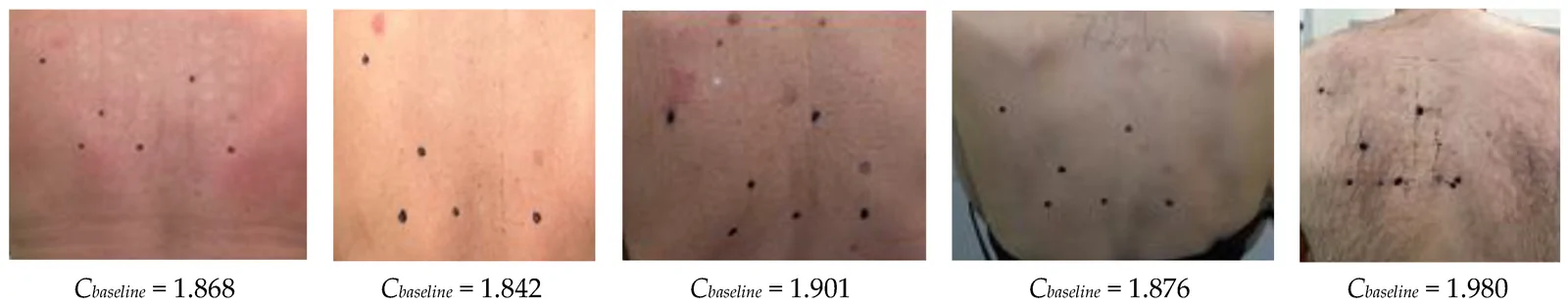
Example images of the tested patients and their corresponding personalized healthy skin baseline coefficients *C_baseline_*, illustrating the natural variance in skin pigmentation across the cohort.

Following the establishment of personalized healthy skin baselines, the soft sensing pipeline evaluated the isolated allergen exposure sites (some examples are presented in Figure 13). The quantitative analysis of the healthy skin coefficient *C_baseline_* revealed significant variations among the subjects. The baseline values ranged from 1.8427 to 1.9853, with the cohort’s overall mean standing at approximately 1.8949. This variance Δ ≈ 0.1426 represents a 7.7% relative difference between the lowest and highest healthy skin baselines, confirming that skin colour characteristics are highly individual and that allergic changes cannot be reliably evaluated by using a single global standard. Because the decision thresholds (*T*_1_ and *T*_2_) were learned strictly during the training phase, the 90% agreement reported in this comparative analysis reflects the algorithm’s performance on a completely independent test cohort, successfully confirming its clinical validity without introducing circular bias.

By calculating the absolute colour deviation *D* = |Δ*C*| for each extracted patch, the system successfully categorized the diverse clinical responses into objective severity classes. The experimental data revealed that allergic contact dermatitis does not manifest uniformly. The measured colour deviations exhibited distinct distributional patterns across the patient cohort. A detailed analysis of specific patient case studies highlights the system’s ability to capture this biological and immunological diversity.

The application of the mathematical thresholds (T1 = 0.05 and T2 = 0.15) to the clinical dataset revealed three primary classification profiles among the patients. These specific values were not chosen arbitrarily, but were statistically established based on the initial visual assessments and annotations provided by the medical expert. We noticed a wide variance in one patient. This case demonstrated a broad and highly variable spectrum of inflammatory responses. The absolute colour deviation values captured a wide range, reaching up to a maximum D of 0.166. The algorithmic classification accurately distributed these reactions into small (54.5%), medium (27.3%), and large (18.2%) categories. Clinically, this wide variance indicates an immune system that reacts with significantly differing intensities to various specific allergens, showcasing the algorithm’s sensitivity across the entire severity spectrum.

In contrast to a broad spectrum, several patients exhibited an exclusively bimodal reaction distribution. The colorimetric analysis categorized exactly 50% of the patches as small reactions and the remaining 50% as large reactions, completely lacking any medium reactions. These cases also recorded one of the highest individual deviations in the dataset, with a maximum deviation of 0.189. This “all-or-nothing” bimodal pattern suggests a highly selective and sharply defined immune response to the tested agents, which the system cleanly segregated without artificially forcing values into the middle tier.

Some patients demonstrated a highly consistent and uniform inflammatory response to the patch test. These patients’ data were overwhelmingly concentrated in a single category. The quantitative analysis resulted in 80% of the tested reactions being classified as medium. While minor deviations existed at the extremes (10% small and 10% large), the dominant clustering of medium-intensity colour shifts indicates a steady, moderate allergic sensitivity across the majority of the applied allergens.

These distinct distributional profiles, ranging from a wide variance to bimodal extremes and moderate consistency, prove that the absolute colour deviation metric *D* is sufficiently dynamic to capture the true heterogeneity of clinical allergic reactions. By automatically translating raw optical data into these structured categories, the proposed soft sensor effectively maps the nuances of complex immune responses, providing a level of objective, quantifiable insight that is difficult to consistently achieve through subjective manual evaluations.

#### 4.2.2. Clinical Evaluation Algorithm vs. Human Dermatologist

**Figure 14:**
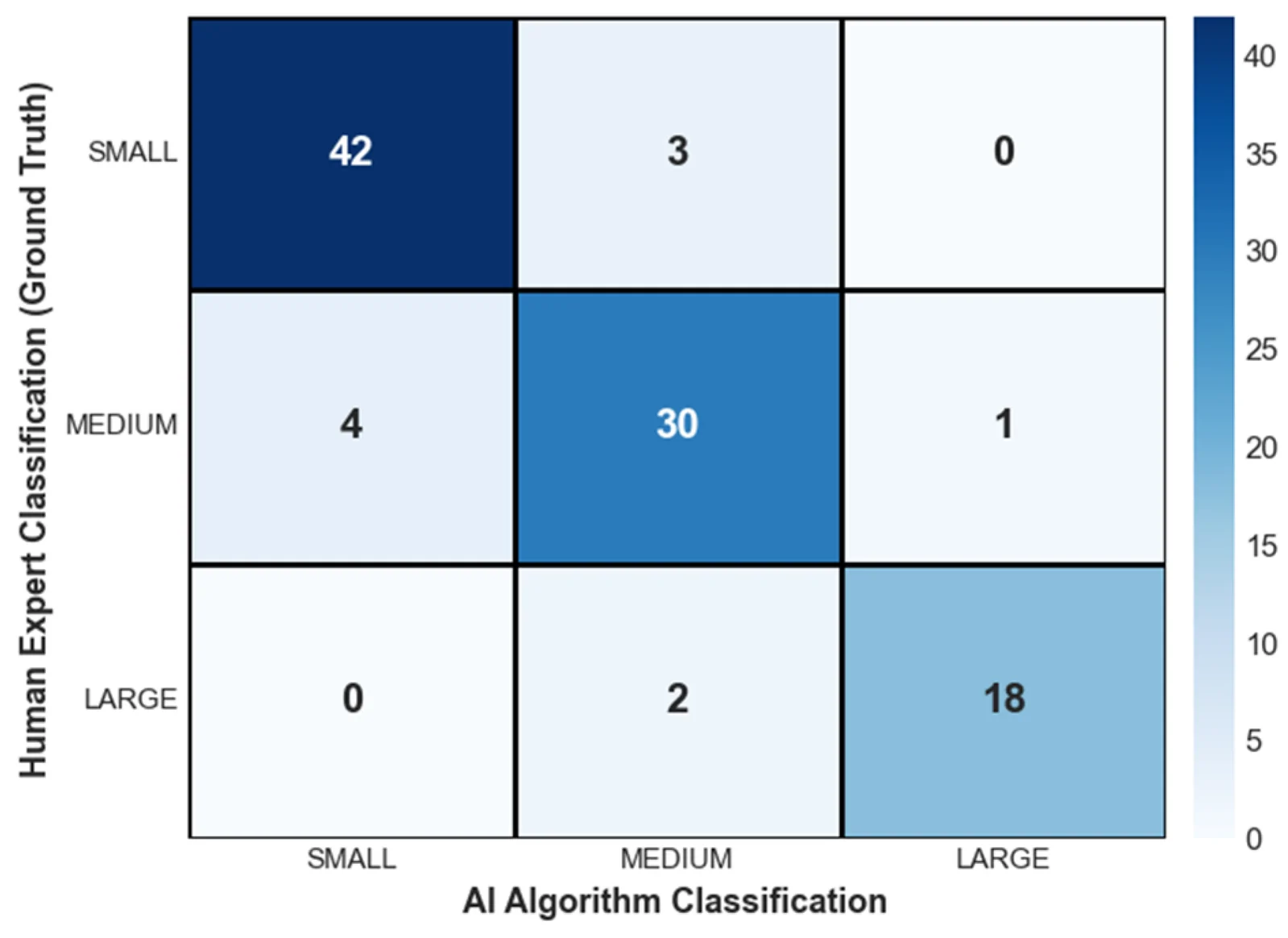
Confusion matrix of proposed algorithm vs. human dermatologist assessment.

Figure 14 represents the confusion matrix that illustrates the performance of a proposed algorithm compared to human expert classifications (ground truth) for evaluating local skin reactions. The matrix evaluates a total of 100 clinical cases distributed across three intensity categories, such as small, medium, and large.

To evaluate the clinical accuracy of the colorimetric threshold classification, the algorithmic outputs were directly compared against the visual assessments of an expert dermatologist. A total of 100 individual patch test reaction cases were selected for this comparative analysis. To ensure an unbiased evaluation of the derived thresholds, these 100 cases were randomly extracted strictly from the independent test cohort, which had never been exposed to the algorithm during the training or threshold formulation phases. The selection process was stratified to ensure a balanced representation across the three severity categories (small, medium, and large), ensuring that the confusion matrix thoroughly stress-tested the algorithm’s performance at the transitional boundaries between grades.

The dark blue diagonal cells represent the instances where the algorithm matched the human dermatologist’s assessment, demonstrating an overall accuracy of 90%. Specifically, the algorithm correctly identified 42 small reactions, 30 medium reactions, and 18 large reactions. To quantify inter-rater reliability beyond random chance, a Cohen’s Kappa coefficient was calculated, yielding κ = 0.84 (95% CI: 0.74–0.94; *p* < 0.001). While this metric indicates substantial agreement between the automated pipeline and the human expert, a comprehensive breakdown of diagnostic performance requires class-specific statistical evaluation. Table 3 details the sensitivity, specificity, precision, and F1-scores for each reaction severity grade, complete with 95% confidence intervals.

The off-diagonal cells, shaded in lighter blue or white, highlight the remaining 10% discrepancy. Notably, all misclassifications occurred exclusively in adjacent transition zones. The algorithm slightly overestimated reactions in four instances (labelling three small cases as medium, and one medium case as large) and underestimated reactions in six instances (labelling four medium cases as small, and two large cases as medium). These specific discrepancies in the medium boundary zones are entirely expected, because in these borderline areas, visual human assessment becomes highly subjective due to subtle erythema (redness) that is difficult to consistently quantify by the naked eye, whereas the algorithm applies strict, mathematically rigid cut-offs.

## 5. Discussion

A fundamental challenge in the automated evaluation of dermatological conditions is the vast natural diversity of human skin pigmentation. Traditional diagnostic systems or basic colorimetric analyses often attempt to apply a universal, fixed threshold to detect erythema or inflammation. However, human skin exhibits significant inter-patient variability due to differing melanin concentrations, baseline vascularity, and individual physiological characteristics. Applying a single global threshold to evaluate allergic contact dermatitis would inevitably lead to systematic classification errors. For instance, a universal threshold might trigger false-positive diagnoses in patients with naturally darker or highly vascularized skin, while simultaneously causing false-negative evaluations in patients with exceptionally pale skin.

The proposed soft sensing pipeline introduces a highly personalized evaluation strategy that establishes a patient-specific reference baseline to overcome this biological variability. By benchmarking allergic reactions against the patient’s own healthy skin, the system successfully categorized reactions with a 90% agreement rate compared to an expert dermatologist. Notably, the 10% discrepancy observed in the confusion matrix occurred exclusively in adjacent transition zones (e.g., confusing a small reaction for a medium one). This indicates that the algorithm behaves safely and predictably. The discrepancies in borderline cases highlight the inherent subjectivity of human visual grading, where slight variations in erythema can lead to different interpretations even among experts, whereas the algorithm strictly adheres to mathematical cut-offs.

Despite its high accuracy, the system has remaining limitations. Visual analysis demonstrated that environmental variables, such as high shadowing caused by natural body contours, the presence of dense body hair, or inconsistent clinical lighting, can still introduce localized segmentation errors. While the CIELAB transformation and baseline normalization mitigate many of these issues, future iterations of the system will require enhanced pre-processing to further suppress artefact interference. Furthermore, because the current findings rely on a single-centre dataset, future multi-centre clinical trials will be necessary to fully validate the system’s generalizability across diverse populations and imaging devices.

## 6. Conclusions

The segmentation results confirm that a hybrid ensemble approach, integrating DeepLabV3+, UperNet, and SegFormer architectures, is highly effective for capturing precise polygonal boundaries in complex medical imagery, outperforming individual standalone models. The ensemble achieved an optimal peak Dice score of 89.3% and an IoU of 81.3%. Furthermore, defining allergic intensity through the absolute colour deviation (*D*) against a patient-specific CIELAB baseline proved to be highly robust, accommodating natural variations in skin pigmentation and lighting conditions. This automated approach achieved a 90% diagnostic agreement with human experts. Future work will focus on integrating skin texture analysis, suppressing environmental artefacts like body hair and shadows, and expanding the dataset to fully operationalize this soft sensor in automated clinical environments.

## Figures and Tables

**Table 1:** Performance comparison of the individual deep learning architectures and the proposed hybrid ensemble.

Model	Dice Score (Target)	Weighted F1-Score	IoU (Target)	AUC
UperNet (Dpn_68)	0.880 (0.867–0.893)	0.902 (0.891–0.914)	0.804 (0.788–0.819)	0.943 (0.931–0.955)
SegFormer (ResNet_34)	0.859 (0.846–0.871)	0.874 (0.862–0.887)	0.768 (0.751–0.785)	0.924 (0.910–0.938)
DeepLabV3+ (Mit_B2)	0.834 (0.819–0.848)	0.853 (0.841–0.866)	0.733 (0.715–0.751)	0.904 (0.888–0.919)
Hybrid Model	0.893 (0.882–0.905)	0.907 (0.895–0.919)	0.813 (0.798–0.827)	0.963 (0.952–0.974)

**Table 2:** Standard F1-score evaluations (macro-, micro-, and class-weighted) across all three semantic classes to ensure statistical transparency.

Model	Macro F1-Score	Micro F1-Score	Class-Weighted F1-Score
UperNet (Dpn_68)	0.931	0.968	0.975
SegFormer (ResNet_34)	0.912	0.953	0.962
DeepLabV3+ (Mit_B2)	0.895	0.941	0.950
Hybrid Model	0.942	0.976	0.982

**Table 3:** Class-specific diagnostic performance metrics comparing the proposed automated evaluation algorithm against expert dermatological assessments, including 95% confidence intervals.

Reaction Grade	Sensitivity (Recall)	Specificity	Precision	F1-Score
Small (+)	93.3% (82.1–98.6%)	92.7% (82.4–98.0%)	91.3% (79.2–97.6%)	92.3% (85.2–96.7%)
Medium (++)	85.7% (69.7–95.2%)	92.3% (83.0–97.5%)	85.7% (69.7–95.2%)	85.7% (74.2–93.4%)
Large (+++)	90.0% (68.3–98.8%)	98.7% (93.2–99.9%)	94.7% (74.0–99.9%)	92.3% (80.1–97.9%)

## Data Availability

The data presented in this study are available on request from the corresponding author.
